# The role of RAD23A in DNA repair and proteasome-dependent protein degradation in multiple myeloma

**DOI:** 10.1038/s41408-026-01574-z

**Published:** 2026-07-15

**Authors:** Xueping Wan, Ting Du, Teng Fang, Minxing Wang, Arghya Ray, Kenneth Wen, Md Abu Musa, Filip Garbicz, Ruben D. Carrasco, Anil A. Samur, Mariateresa Fulciniti, Srikanth Talluri, Yan Song, Nikhil C. Munshi, Kenneth C. Anderson

**Affiliations:** 1 LeBow Institute for Myeloma Therapeutics and Jerome Lipper Myeloma Center, Department of Medical Oncology, Dana-Farber Cancer Institute, Harvard Medical School, Boston, MA USA; 2 Department of Oncologic Pathology, Dana-Farber Cancer Institute, Harvard Medical School, Boston, MA USA; 3 VA Boston Healthcare System, Boston, MA USA

**Keywords:** Targeted therapies, Myeloma

## Abstract

Multiple myeloma (MM) is a plasma cell malignancy characterized by genomic instability and a high dependency on proteasome activity. Accordingly, DNA-damaging agents and proteasome inhibitors (PIs) are frontline therapies; however, therapeutic resistance inevitably develops. RAD23A, a dual-function protein involved in nucleotide excision repair and proteasome-mediated protein degradation, is highly expressed in MM and is associated with poor patient survival. Here, we demonstrate that depletion of RAD23A exerts potent anti-MM activity both in vitr*o* and in *vivo*. Inducible CRISPR/Cas9 knockout (iKO) or shRNA-mediated knockdown of RAD23A in MM cell lines, including PI-resistant models, markedly suppressed cell proliferation. RAD23A loss impaired DNA repair capacity, triggered activation of the stress-associated eIF2α–ATF4 axis and promoted caspase-dependent apoptosis. Quantitative proteomic profiling of RAD23A-iKO cells confirmed upregulation of apoptotic and stress-response pathways, accompanied by downregulation of DNA repair and deubiquitination pathways. In an MM xenograft model, RAD23A depletion significantly reduced tumor growth and prolonged survival. Collectively, these findings identify RAD23A as a critical survival factor in MM and highlight its potential as a novel therapeutic target.

## Introduction

Multiple myeloma (MM) is a plasma cell malignancy and is the second most common hematological cancer worldwide [1]. Multiple myeloma cells are characterized by extensive DNA damage accompanied by dysregulated DNA repair pathways, which contribute to disease progression, clonal evolution, and therapeutic resistance [2]. In parallel, the excessive production of immunoglobulins imposes a substantial proteotoxic burden, rendering MM cells heavily dependent on the ubiquitin-proteasome system (UPS) for survival [3]. Therefore, DNA-damaging chemotherapeutics, such as melphalan, and proteasome inhibitors (PIs), such as bortezomib, have become cornerstones of standard MM therapy [4–6]. Despite significant clinical benefit, most patients ultimately develop resistance to these agents, which remains the primary challenge to long-term survival [7]. Identifying novel therapeutic targets that intersect these fundamental pathways is therefore essential for overcoming treatment resistance in MM.

RAD23A, also known as human RAD23 homolog A (hHR23A), is one of the human orthologs of yeast RAD23 protein [8]. It contains four functional domains: an N-terminal ubiquitin-like (UBL) domain, a xeroderma pigmentosum group C (XPC)-binding domain, and two ubiquitin-associated domains (UBA1 and UBA2) [9]. Through these domains, RAD23A plays dual roles in both the nucleotide excision repair (NER) and the UPS pathways [10]. In the context of DNA repair, RAD23A binds to XPC, serving as a key component for detecting and repairing DNA damage during the early steps of NER [11]. As a ubiquitin shuttle factor, RAD23A facilitates the delivery of substrates to the 26S proteasome by binding polyubiquitinated substrates via its UBA domains and interacting with the PSMD4 subunit on the proteasome through its UBL domain [9].

RAD23A dysregulation has been implicated in multiple human diseases. In amyotrophic lateral sclerosis, RAD23A acts as a significant modifier of protein toxicity, and therapeutic strategies using antisense oligonucleotides or genetic knockdown have demonstrated remarkable efficacy in preclinical models, leading to significant lifespan extension and reduced protein aggregation through improved UPS function [12]. In cancer, RAD23A has been identified as a potential prognostic biomarker for high-risk acute myeloid leukemia [13], and transcriptomic profiling in MM had identified RAD23A as a strong predictor of high-risk disease [14]. Together, these findings position RAD23A as a biologically and clinically significant target in disease pathogenesis, providing a compelling rationale to dissect its functional role in MM.

In this study, we investigated the effects of RAD23A depletion in MM cells and murine xenograft models. We demonstrate that genetic depletion of RAD23A induces DNA damage, disrupts proteasome-mediated protein degradation, activates stress signaling pathways, and triggers apoptosis, resulting in pronounced anti-tumor activity, including in PI-resistant MM models. Collectively, our findings suggest that targeting RAD23A may overcome therapeutic resistance, improve clinical outcomes for MM patients, and establish RAD23A as a novel therapeutic vulnerability in MM.

## Methods

### Cell culture

MM cell lines were cultured in RPMI 1640 medium (Gibco, Thermo Fisher Scientific) supplemented with 10% fetal bovine serum (Gibco) and 1% penicillin/streptomycin (Gibco). MM patient CD138^+^ cells and CD138^-^ cells were isolated as previously described [15]. Informed consent was obtained from all patients in accordance with the Declaration of Helsinki. All cells were incubated in a standard humidified incubator maintained at 37 °C with 5% CO_2_.

### Western blotting

Cells were lysed in RIPA buffer supplemented with a complete protease inhibitor cocktail (Cell Signaling Technology, #5871). Protein concentrations were determined using a protein assay dye (Bio-Rad, #5000006). Equal amounts of protein (10 µg) were separated by SDS-PAGE and transferred to PVDF membranes using a semi-dry transfer system iBlot3 (Thermo Fisher Scientific). Membranes were blocked and incubated with primary antibodies overnight at 4 °C, followed by incubation with HRP-conjugated secondary antibodies. Protein bands were detected using enhanced chemiluminescence (Bio-Rad, #1705061), and images were acquired with the ImageQuant 800 system (Cytiva Amersham). Primary antibodies against K48-linked polyubiquitin (#12805S), p21(#2947S), phospho-CHK2 (Thr68; #2197S), PARP (#9542S), cleaved caspase-3 (#9661S), phospho-eIF2α (Ser51; #9721S), ATF4 (#11815S) and CHOP (#2895S) were purchased from Cell Signaling Technology. Antibodies against RAD23A (#sc-365669) and β-actin (C4) HRP (#sc-47778) were purchased from Santa Cruz Biotechnology. Antibody against γ-H2AX (#MA1-2022) was purchased from Thermo Fisher Scientific.

### Generation of inducible RAD23A knockdown and knockout cell lines

To generate inducible RAD23A knockdown (RAD23A-iKD) cell lines, H929, ANBL6-WT and bortezomib-resistant ANBL6-BR cell lines were transduced with a lentiviral vector encoding RAD23A-specific short hairpin RNA (shRNA) under a doxycycline (Dox)-inducible promoter (Horizon Discovery). Positive cells were selected with puromycin for 5 days. RAD23A knockdown was induced with 1 µg/ml Dox, which was administered every other day.

Inducible RAD23A knockout (RAD23A-iKO) cell lines were generated in AMO1 and MM.1R cells using a Dox-inducible CRISPR/Cas9 system. Briefly, cells were first transduced with a lentivirus expressing Dox-inducible Cas9 (GeneCopoeia) and selected with G418 to establish inducible Cas9-expressing cell populations. Following selection, cells were transduced with a second lentivirus encoding a single-guide RNA (sgRNA) targeting RAD23A. Cells were then selected with puromycin for 7 days. Cas9 expression was induced with Dox (1.5 µg/ml for AMO1 and 1 µg/ml for MM.1R), which was administered every other day. RAD23A knockout or knockdown efficiency was confirmed by western blot analysis. To generate stable RAD23A add-back cells, lentivirus-packaged Flag-tagged RAD23A or empty vector plasmids were transfected into MM.1R RAD23A-iKO cells, and positive cells were selected with blasticidin.

### Cell proliferation assay

MM cell proliferation was monitored using the IncuCyte SX5 live-cell imaging system (IncuCyte 2025, Sartorius). Briefly, 5000–10,000 cells per well were seeded in 96-well clear flat-bottom plates (Corning, #353072). For suspension cells, plates were pre-coated with Poly-D-lysine (Sigma-Aldrich, #A-003-E) to facilitate cell attachment. Following seeding, plates were placed in the IncuCyte system and maintained at 37°C with 5% CO_2_. Phase-contrast images were acquired at 1-day intervals over the indicated time course. Cell proliferation was quantified using IncuCyte software by measuring cell confluence over time and was normalized to the initial time point for each condition. Data are presented as fold change relative to the initial time point.

### Quantitative proteomic analysis

Quantitative proteomic analysis was performed on AMO1 RAD23A-iKO and control cells following 4 days of Dox treatment with triplicates for each condition. Global proteomic profiling was conducted using isobaric tandem mass tag (TMT) labeling. Detailed procedures for sample preparation and mass spectrometry analysis have been described previously [16]. Briefly, cells were harvested, washed with cold PBS, and lysed for protein extraction and quantification. Proteins were subsequently reduced, digested, and labeled with TMT reagents prior to mass spectrometry analysis. Peptides and proteins were filtered at an FDR < 1%. Only proteins identified with more than one unique peptide were included in downstream analysis. Differential protein expression was analyzed using the limma package in R [17], with adjustment for multiple hypotheses using the Benjamini-Hochberg method. Proteins with *adjusted p* < 0.05 and fold change > 1.5 were considered differentially expressed. Gene set enrichment analysis (GSEA) was performed using curated gene sets from the Molecular Signatures Database (MSigDB) [18, 19], including the Reactome and Hallmark collections [20, 21]. Gene sets with at least 50 matched genes were included in the analysis. Visualization, including the volcano plot and scatter plot, was conducted with matplotlib and seaborn packages in Python 3 [22, 23].

### Immunofluorescence and microscopy

2 × 10^5^ cells were seeded into 96-well glass-bottom plates (Cellvis, #NC0536760). Plates were pre-coated with poly-D-lysine (Sigma-Aldrich, #A-003-E) prior to cell seeding. Immunofluorescence staining was performed with minor modifications to previously published protocols [24]. Briefly, cells were washed with cold PBS and fixed with 4% (v/v) paraformaldehyde at room temperature (RT) for 10 min, followed by permeabilization with 0.5% Triton X-100 for 15 min. Cells were then blocked with 5% BSA for 60 min at RT and incubated overnight at 4 °C with the following primary antibodies: γ-H2AX (Sigma-Aldrich, #05-636-I) or RAD51 (Abcam, #ab63801). After primary antibody incubation, cells were washed with PBS and incubated with secondary antibodies, namely the Alexa Fluor 488 goat anti-mouse IgG (Thermo Fisher Scientific, #A-11001) and Alexa Fluor 488 goat anti-rabbit IgG (Thermo Fisher Scientific, #A-11008) for 60 min at RT in the dark. Samples stained with γ-H2AX were covered with PBS and imaged immediately using the IncuCyte system. Samples stained with RAD51 were counterstained and mounted with ProLong Diamond Antifade Mountant with 4’,6-diamidino-2-phenylindole (DAPI) (Thermo Fisher Scientific, #P36962). Images were acquired using a confocal microscope (Zeiss 980) and analyzed using ImageJ software (National Institutes of Health).

To detect K48-linked polyubiquitin and aggresome, immunostaining was performed with minor modifications to the protocol described above. Briefly, following fixation, permeabilization, and blocking, samples were incubated overnight at 4 °C with Alexa Fluor 647 Anti-ubiquitin (linkage-specific K48) (Abcam, #ab204476). Cells were then washed with PBS and incubated with aggresome detection reagent (Enzo Life Sciences, #ENZ-51035) for 30 min at RT. Samples were counterstained, mounted, and imaged as described above for RAD51 imaging.

### DNA slot blot repair assay

NER activity was assessed using a DNA slot blot repair assay as previously described [25], with minor modifications. Briefly, equal numbers of cells were seeded in 60-mm culture dishes and cultured overnight. Cells were then exposed to UV-C irradiation (10 J/m^2^) and collected at the indicated time points following UV treatment. Genomic DNA was isolated using a commercial DNA extraction kit (Thermo Fisher Scientific, #K182002) and quantified using a NanoDrop spectrophotometer (Thermo Fisher Scientific).

For each sample, 500 ng of genomic DNA was diluted to a final volume of 300 µL in nuclease-free water, denatured by boiling for 10 min, and immediately placed on ice. Denatured DNA (300 μL) was loaded onto a nitrocellulose membrane using a Manifold II Slot-Blot System (Bio-Dot Apparatus, #1706545). The membrane was air-dried overnight at RT to immobilize the DNA. The membrane was then washed with TBST for 10 min and blocked with 5% non-fat dry milk in TBS, followed by incubation with an anti-(6-4) photoproduct [(6-4)PP] antibody (Cosmo Bio, #CAC-NM-DND-002) overnight at 4 °C. After washing, the membrane was incubated with an HRP-conjugated anti-mouse secondary antibody (Cell Signaling Technology, #7076S) for 1 h at RT. Signals were detected using enhanced chemiluminescence and visualized with an ImageQuant 800 system. As a loading control, the membrane was stained with SYBR Gold nucleic acid dye (Thermo Fisher Scientific, #S11494) for 20 min in the dark, washed with TBST, and imaged using the same detection system.

### Proteasome activity assays

Proteasome activity assays were performed as previously reported [26]. MM.1R control (CT) and RAD23A-iKO cells, as well as MM.1R cells treated with DMSO or 1 µM bortezomib for 3 h, were harvested and lysed. Following centrifugation, protein supernatants were collected for analysis. Proteasome activity was measured using the Proteasome 20S Assay Kit (Enzo Life Sciences, #BML-AK740) according to the manufacturer’s instructions. A total of 24 µg of protein was used per reaction.

### Apoptosis assays

Caspase-3/7-mediated apoptosis was assessed in MM.1R control and RAD23A-iKO cells using the IncuCyte system. Cells were cultured in 96-well plates in the presence of Dox. The IncuCyte Caspase-3/7 Dye (Sartorius, #4440) was added directly to the culture medium according to the manufacturer’s instructions, and apoptosis-associated fluorescence signals were monitored over time using the IncuCyte system.

Apoptosis was further validated by flow cytometric analysis using an Annexin V-FITC/DAPI apoptosis detection kit (Elabscience, #E-CK-A252), following the manufacturer’s protocol. Briefly, 1 × 10^6^ cells were washed with PBS and resuspended in 100 μL of 1 × binding buffer. Cells were then incubated with 2.5 µL of Annexin V-FITC/DAPI for 30 min at RT in the dark. After incubation, 400 µL of 1 × binding buffer was added, and samples were analyzed within an hour using a BD flow cytometer (BD Biosciences). Data were analyzed using FlowJo software (FlowJo LLC, BD Biosciences).

### MM xenograft model

Six-week-old female NSG mice were randomized into experimental groups based on body weight to ensure comparable average body weights across groups prior to tumor inoculation. Mice were subcutaneously inoculated with 4.0 × 10^6^ MM.1R control cells, RAD23A-iKO cells, or RAD23A-iKO add-back cells. Investigators were not blinded to group allocation during the animal experiments. Three days after inoculation, mice were placed on an irradiated Dox-containing diet (0.0625% Dox) to induce RAD23A knockout. Tumor size was measured every other day using a digital caliper. Tumor volume was calculated using the formula: (a × b^2^) / 2, where *a* represents the longest tumor diameter and *b* represents the shortest tumor diameter. Mice were euthanized once tumor volume reached the institutional endpoint of 2000 mm^3^.

All animal experimental protocols were approved by and conformed to the relevant regulatory standards of the Institutional Animal Care and Use Committee at the Dana-Farber Cancer Institute.

### Statistical analysis

Statistical significance was determined by the tests noted in the figure legends. All data were analyzed using GraphPad Prism 11 software (GraphPad, Boston, MA). *p ≤ 0.05, ** *p* ≤ 0.01, *** *p* ≤ 0.001, ****p ≤ 0.0001.

## Results

### Expression and clinical significance of RAD23A in MM

**Fig. 1:**
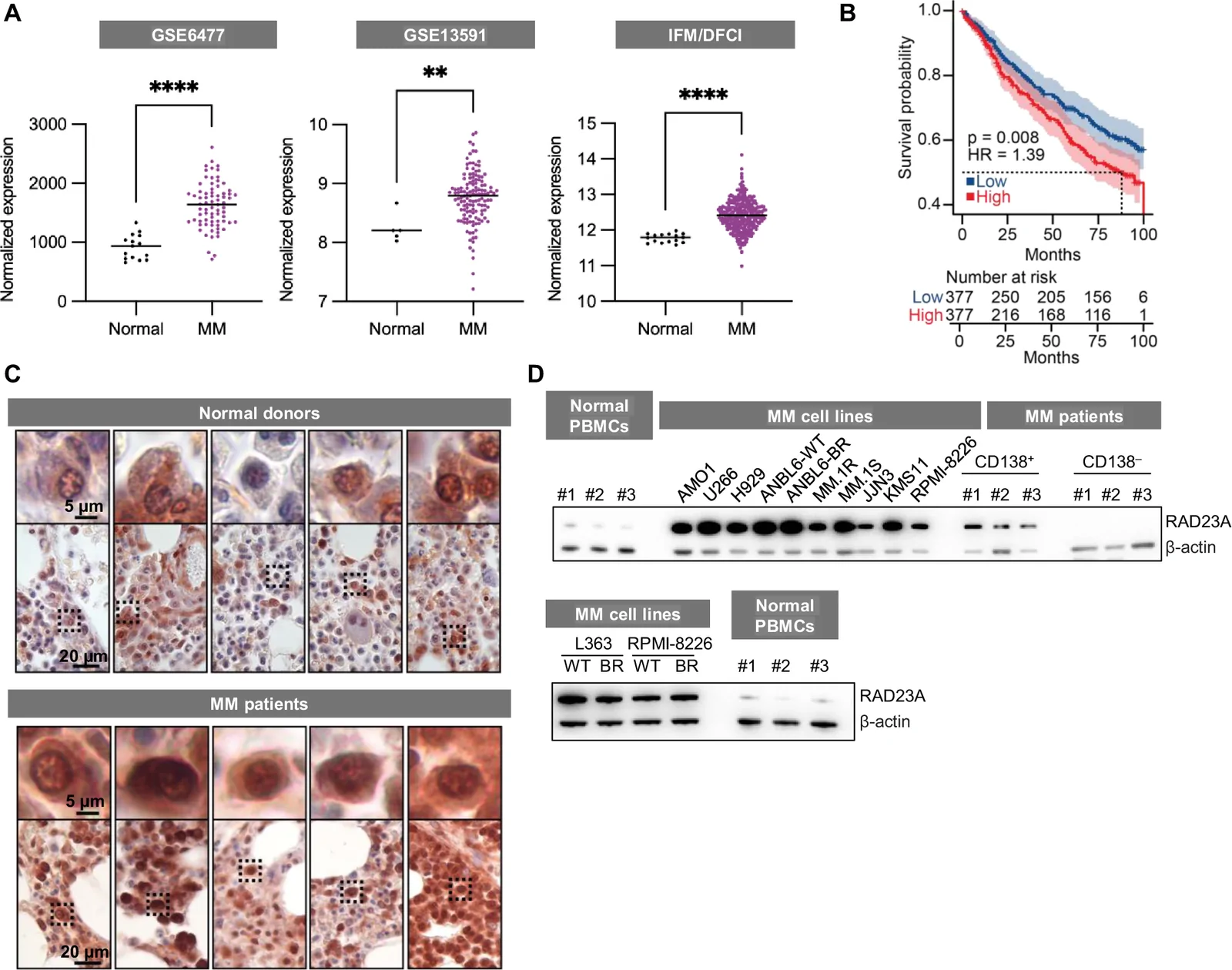
RAD23A expression and prognostic significance in MM. **A** *RAD23A* expression was analyzed using publicly available datasets (GSE6477 and GSE13591) and the internal IFM/DFCI MM database. GSE6477 includes 15 normal and 73 MM samples; GSE13591 includes 5 normal and 133 MM samples; and the IFM/DFCI database contains 16 normal and 319 MM samples. Significance was determined by Welch’s t-test, with the median marked by a line. **B** Kaplan-Meier analysis of *RAD23A* expression and overall survival in MM patients. A total of 754 samples from the CoMMpass-MMRF RNA-seq dataset (MMRF_IA22) were included. Patients were grouped into high- and low-expression groups based on the median *RAD23A* expression level. Statistical significance was assessed using the log-rank test. **C** Immunohistochemical analysis of RAD23A expression in bone marrow biopsies from normal donors and MM patients. Plasma cells are marked by dotted lines and are shown in the corresponding zoomed-in images. **D** Immunoblot analysis of RAD23A and β-actin in protein lysates from MM cell lines, normal PBMCs, and CD138^+^ or CD138^-^ cells isolated from MM patients.

We first analyzed two publicly available datasets, GSE6477, GSE13591, and our IFM/DFCI cohort [27], and found that *RAD23A* expression was significantly higher in MM cells from patients compared with normal plasma cells (Fig. 1A). Furthermore, higher *RAD23A* expression in CD138^+^ bone marrow cells was associated with significantly shorter overall survival in 754 MM patients (CoMMpass_IA22 cohort) (Fig. 1B). Immunohistochemical studies confirmed elevated RAD23A expression in bone marrow biopsies from MM patients compared with healthy donors (Fig. 1C). Consistent with these results, immunoblot analysis showed increased RAD23A protein levels in MM cell lines compared with normal PBMCs and in primary CD138^+^ tumor cells compared with paired CD138^-^ cells isolated from MM patients (Fig. 1D).

### Functional role of RAD23A in vitro and in vivo

**Fig. 2:**
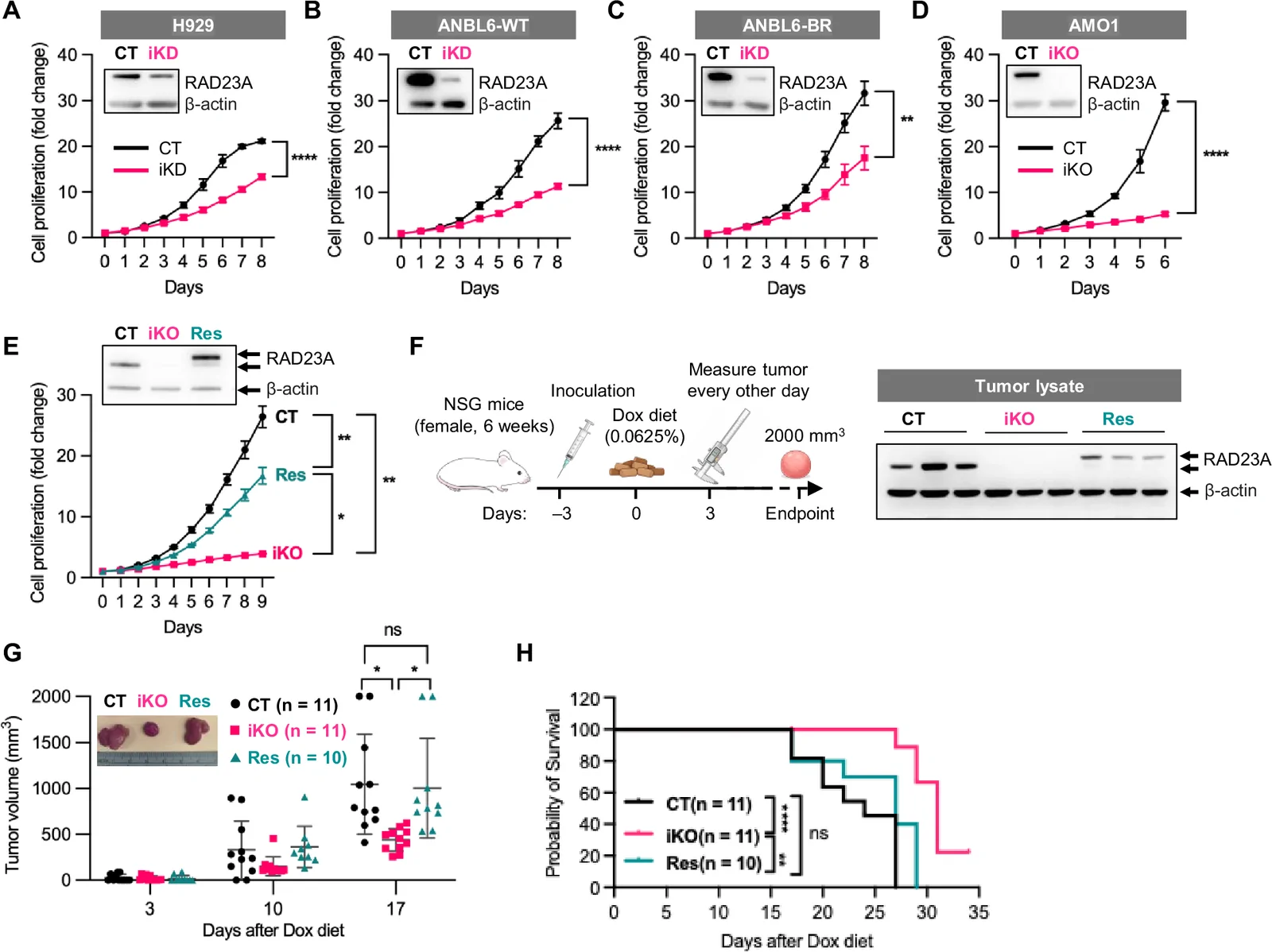
Functional significance of RAD23A. **A**–**C** Proliferation of H929, ANBL6-WT, and ANBL6-BR cells with inducible RAD23A shRNA knockdown (iKD) was monitored using the IncuCyte live-cell imaging system (mean ± SD, *n* = 3, one of three independent experiments is shown, significance assessed with two-way repeated measures ANOVA). Doxycycline (Dox; 1 µg/ml) was added every two days. Immunoblotting confirmed RAD23A knockdown. CT = control. **D** Proliferation of AMO1 cells with inducible RAD23A knockout (iKO) was monitored by IncuCyte (mean ± SD, *n* = 3, one of three independent experiments is shown, significance assessed with two-way repeated measures ANOVA). Dox (1.5 µg/ml) was added every other day. Immunoblotting shows RAD23A protein levels. **E** Proliferation of MM.1R RAD23A-iKO cells and RAD23A add-back cells (RAD23A-iKO cells transduced with lentiviral Flag-RAD23A or empty vector, followed by blasticidin selection) was monitored using IncuCyte (mean ± SD, *n* = 3, one of three independent experiments is shown, significance assessed with two-way repeated measures ANOVA with Sidak’s test). Only the significance of the last time point is shown. Dox (1 µg/ml) was added every two days. Immunoblotting confirmed RAD23A expression. Res = Rescue. **F** NSG mice were subcutaneously inoculated with MM.1R control, RAD23A-iKO or RAD23A add-back (Res) cells. RAD23A knockout was induced by administration of a Dox-containing diet (irradiated 0.0625% Dox; ~1-6 mg per mouse per day) starting 3 days after inoculation. Left: schematic of the xenograft experimental design. Right: Immunoblot analysis of RAD23A expression in xenograft tumor lysates. **G** Tumor volumes (mm^3^) are shown as mean ± SD over time and were analyzed with a mixed-effects model with Tukey’s test. Only the significance for the last time point is shown. (CT, *n* = 11; iKO, *n* = 11; Res, *n* = 10). Representative tumor images were collected 17 days after initiation of Dox diet. **H**. Kaplan-Meier survival analysis of mice; statistical significance was determined using a log-rank (Mantel-Cox) test performed in GraphPad Prism (CT, *n* = 11; iKO, *n* = 11; Res, *n* = 10).

To investigate the functional role of RAD23A in MM, we first evaluated the effects of inducible RAD23A knockdown using shRNA. Dox-induced downregulation of RAD23A significantly inhibited cell growth in H929, ANBL6-WT and bortezomib-resistant ANBL6-BR cell lines (Fig. 2A–C), indicating that RAD23A is required for MM cell proliferation irrespective of bortezomib sensitivity. We next generated inducible CRISPR/Cas9-mediated RAD23A knockout cells in the AMO1 and MM.1R cell lines. Consistent with the shRNA results, Dox-induced RAD23A depletion significantly inhibited cell growth in both AMO1 and MM.1R cells (Fig. 2D, E). Importantly, re-expression of RAD23A in MM.1R RAD23A-iKO cells partially restored cell growth (Fig. 2E), confirming that the observed growth inhibition was specifically attributable to RAD23A loss.

To validate the anti-myeloma effect of RAD23A depletion in vivo, we established a subcutaneous xenograft mouse model using MM.1R control, RAD23A-iKO and RAD23A add-back cell lines (Fig. 2F–H). Consistent with the in vitro results, RAD23A knockout markedly reduced tumor growth and prolonged survival. While re-expression of RAD23A restored tumor growth, confirming the specificity of the effects. Collectively, these findings demonstrate that RAD23A is critical for MM tumor growth and highlight RAD23A as a potential therapeutic target in MM.

### Proteomic profiling of RAD23A depletion

**Fig. 3:**
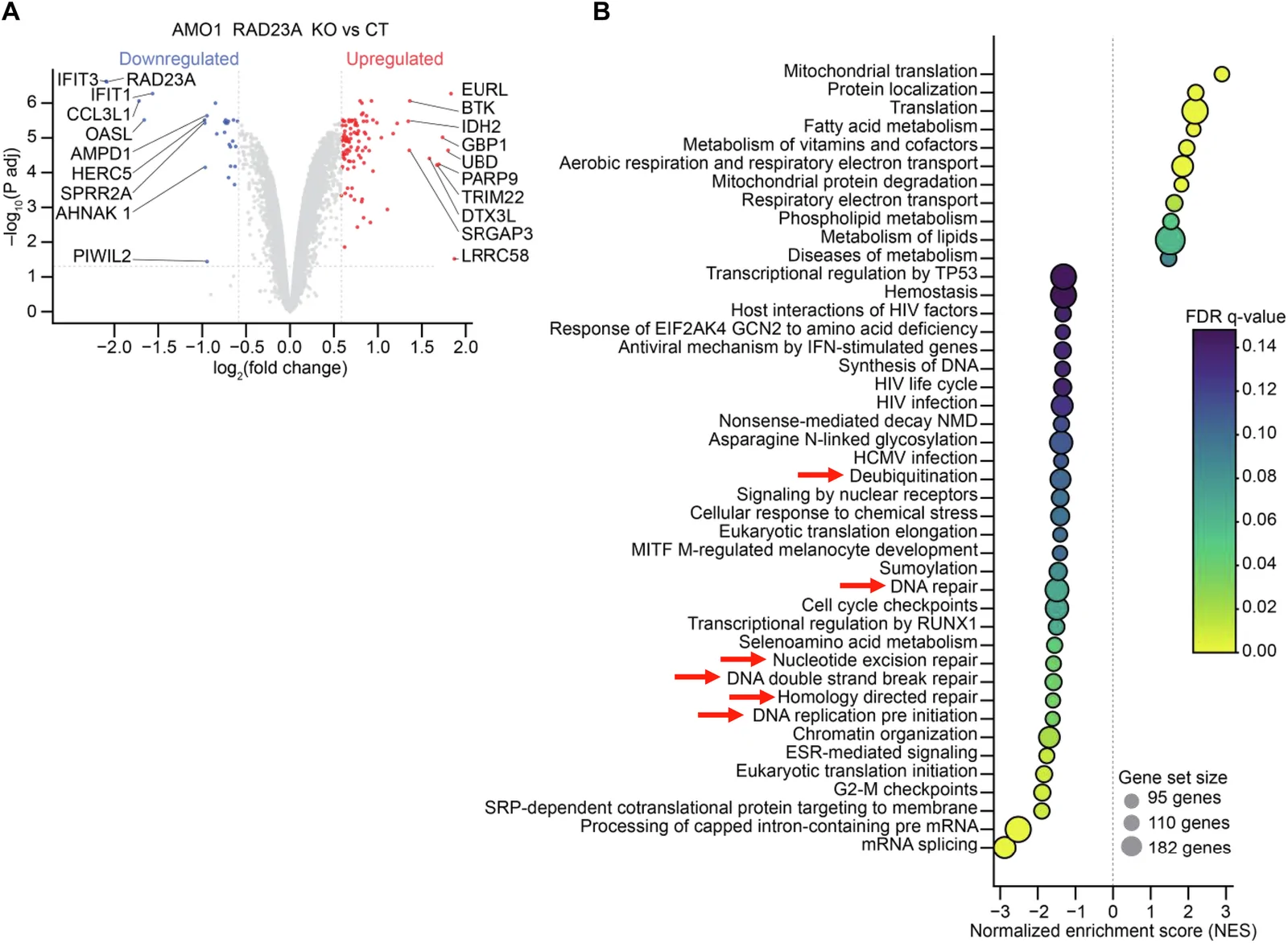
Proteomic analyses of RAD23A knockout in MM. **A** Volcano plot showing differentially expressed proteins in RAD23A-iKO cells compared with control cells, as determined by TMT-based quantitative proteomics. The top 10 proteins with the most significant changes in abundance are labelled (fold change > 1.5, adjusted *p* < 0.05). **B** Gene set enrichment analysis based on the Reactome database demonstrating enrichment of DNA repair- and proteasome-related pathways among downregulated proteins, and mitochondrial- and metabolism-related pathways among upregulated proteins in RAD23A-depleted cells. Significantly enriched gene sets are defined by a cutoff of FDR *q* < 0.15 and gene set size > 80.

To obtain mechanistic insights into the anti-tumor effects of RAD23A depletion in MM, we performed tandem mass tag (TMT)-based quantitative proteomic analysis using AMO1 RAD23A-iKO cells. A total of 6902 high-confidence proteins were quantified and included in downstream bioinformatic analysis. In RAD23A-iKO cells, 104 proteins were significantly upregulated, and 28 proteins were significantly downregulated compared with control cells (fold change > 1.5, adjusted *p* < 0.05) (Fig. 3A). Gene set enrichment analysis (GSEA) using the Reactome gene sets showed that DNA repair-related and proteasome-related pathways were enriched among the downregulated proteins, whereas mitochondrial-related and metabolism-related pathways were enriched among the upregulated proteins in RAD23A-depleted cells (Fig. 3B).

### RAD23A regulates DNA damage repair in MM

**Fig. 4:**
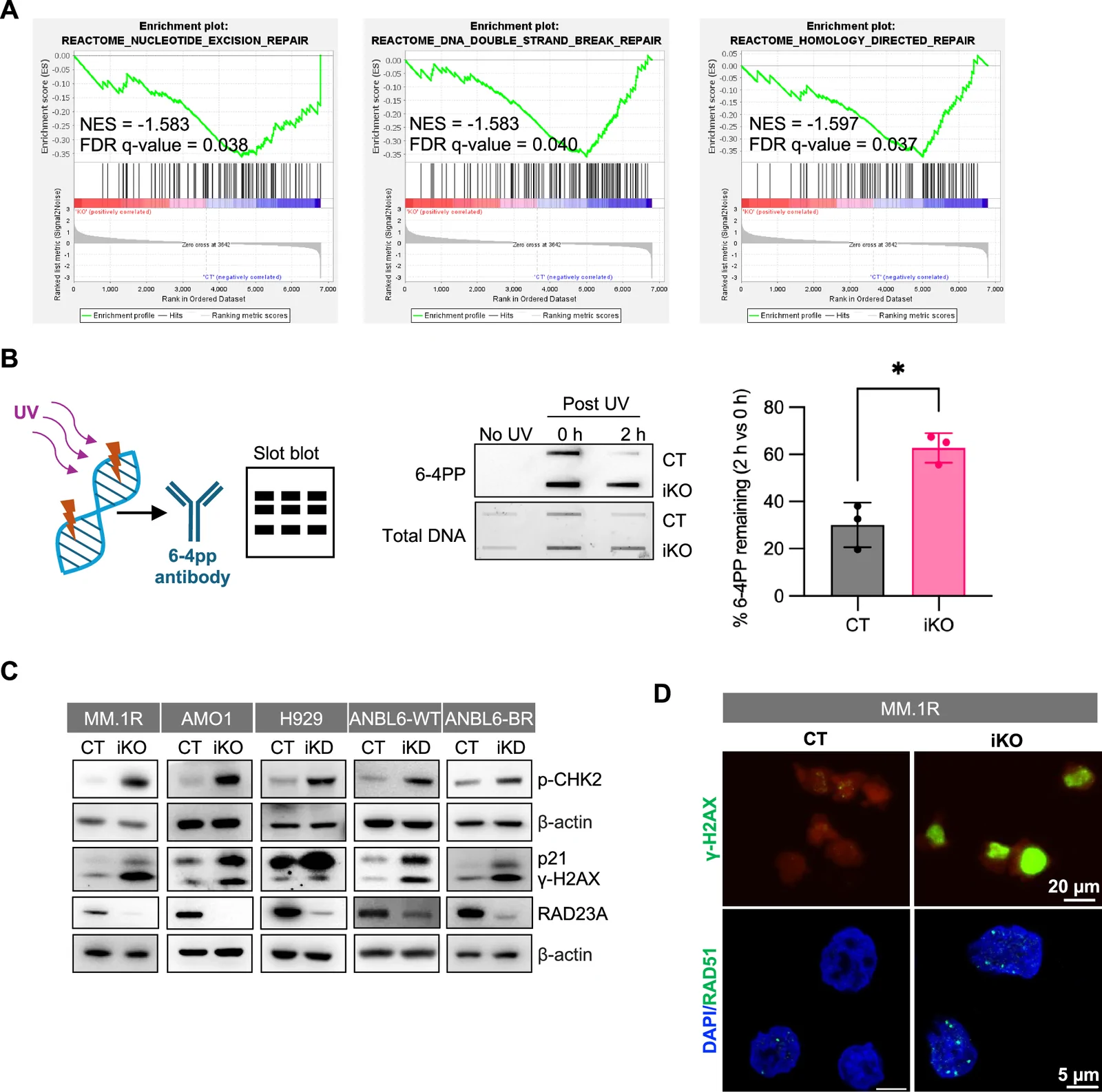
RAD23A regulates DNA damage repair in MM cells. **A** Gene set enrichment analysis of Reactome DNA repair pathways following RAD23A depletion shows significant negative enrichment. Enrichment plots include NER (left, NES = −1.583, FDR *q*-value = 0.038), DNA double-strand break repair (middle, NES = −1.583, FDR *q*-value = 0.04), and homology-directed repair (right, NES = −1.597, FDR *q*-value = 0.037). **B** MM.1R RAD23A-iKO cells were cultured with Dox for 9 days, then exposed to UV and harvested at the indicated time points. Genomic DNA was extracted, and UV-induced 6-4 photoproducts [(6-4)PP] were detected by slot blot analysis using a specific antibody. SYBR Gold staining was used to detect total DNA. Left, schematic of slot blot assay. Middle, representative slot blot showing (6-4)PP and loading control. Right, quantification of remaining (6-4)PP lesions, calculated by normalizing the (6-4)PP signal to total DNA (mean ± SD, *n* = 3, analyzed by Welch’s t-test). CT = control. **C** MM.1R and AMO1 RAD23A-iKO cells (cultured with Dox for 9 and 4 days, respectively), as well as H929, ANBL6-WT, and ANBL6-BR cells with RAD23A-iKD (cultured with Dox for 6 days), were analyzed by immunoblotting using antibodies against p-CHK2, γ-H2AX, p21, RAD23A, and β-actin. **D** MM.1R RAD23A-iKO cells were cultured with Dox for 9 days, then fixed and stained with γ-H2AX and RAD51 antibodies and visualized by IncuCyte (20×) or confocal microscopy (Zeiss 980, 60×). Above, γ-H2AX is shown in green. Below, nuclei are shown in blue, and RAD51 foci are shown in green.

To investigate the role of RAD23A in DNA repair in MM cells, we performed GSEA focusing on DNA repair-related pathways using proteomic data from AMO1 RAD23A-iKO cells. Notably, downregulated proteins were significantly enriched in the NER (NES = −1.583, FDR *q* = 0.038), DNA double-strand break repair (NES = −1.583, FDR *q* = 0.04), and homology-directed repair (NES = −1.597, FDR *q* = 0.037) pathways (Fig. 4A), indicating a global impairment of DNA repair capacity upon RAD23A loss.

We next directly assessed NER efficiency by quantifying the amount of unrepaired (6-4) photoproduct [(6-4)PP] following UV exposure. MM.1R RAD23A-iKO cells exhibited significant accumulation of unrepaired lesions compared with control cells, indicating compromised NER activity upon RAD23A depletion (Fig. 4B). Consistent with the proteomic analysis, immunoblot analysis confirmed enhanced DNA damage signaling in RAD23A-depleted MM cells, as evidenced by increased expression of p-CHK2, γ-H2AX, and p21 (Fig. 4C). Furthermore, immunofluorescence analysis revealed elevated γ-H2AX signal and increased RAD51 foci formation in RAD23A-iKO cells, reflecting accumulation of unrepaired DNA damage (Fig. 4D). Together, these results support a critical role for RAD23A in maintaining DNA repair capacity and genome stability in MM.

### RAD23A modulates proteasome function and stress response pathways in MM

**Fig. 5:**
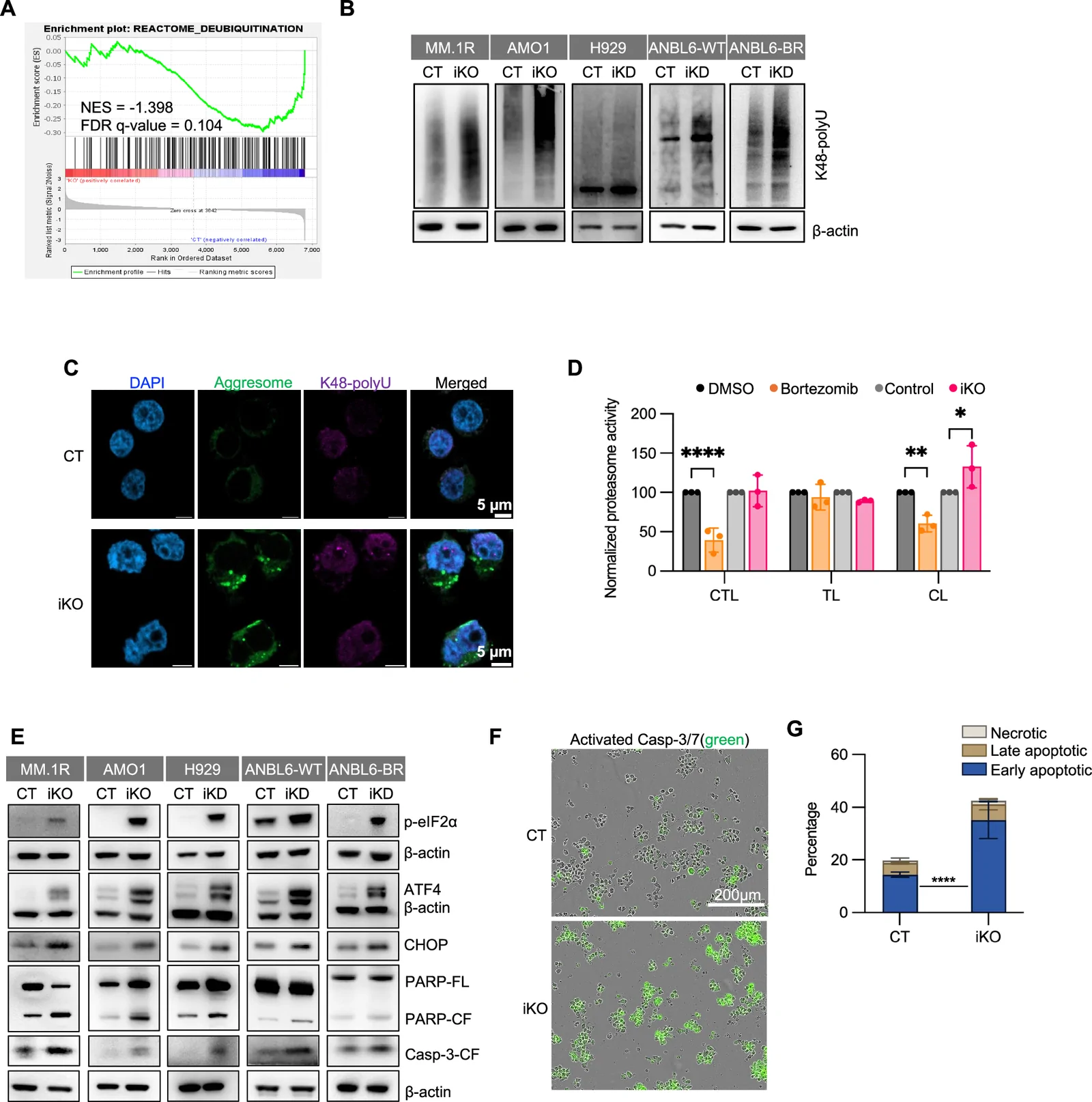
RAD23A depletion impairs protein degradation and induces stress responses. **A** Proteomic-based GSEA Reactome showing negative enrichment of the deubiquitination pathway following RAD23A knockout (NES = −1.398, FDR *q*-value = 0.104). **B** MM.1R and AMO1 RAD23A-iKO cells, as well as H929, ANBL6-WT, and ANBL6-BR RAD23A-iKD cells, were cultured with Dox as described in Fig. 4C. Protein lysates were subjected to immunoblotting using antibodies against K48-linked polyubiquitin and β-actin. CT = control. **C** Aggresome and K48-linked polyubiquitinated protein (K48-polyU) accumulation were detected using an aggresome detection kit and anti-K48-polyubiquitin antibody, followed by confocal microscopy (60×). **D** MM.1R RAD23A-iKO cells were cultured with Dox for 9 days. MM.1R cells treated with DMSO or 1 µM bortezomib for 3 h were used as controls. Cell pellets were harvested and lysed. 20S proteasome activities (CTL, chymotrypsin-like; TL, trypsin-like; CL, caspase-like) were measured. Proteasome activity was normalized to the DMSO control or parental MM.1R control cells (mean ± SD, *n* = 3, assessed by two-way ANOVA with Tukey’s test). **E** MM.1R and AMO1 RAD23A-iKO cells, as well as H929, ANBL6-WT and ANBL6-BR RAD23A-iKD cells, were prepared as described in Fig. 4C. Cells were lysed and immunoblotted using antibodies against p-eIF2α, ATF4, CHOP, PARP, cleaved caspase-3, and β-actin. **F** Caspase-3/7 activation was detected using the IncuCyte Caspase-3/7 Green Dye and live-cell imaging. **G** Apoptosis was analyzed by flow cytometry using Annexin V-FITC/DAPI double staining (mean ± SD, *n* = 3, assessed by two-way ANOVA with Sidak’s test).

We next investigated whether RAD23A depletion disrupts proteasome function in MM cells. Proteomics-based GSEA revealed significant downregulation of proteasome-related pathway following RAD23A knockout, with the deubiquitination pathway showing negative enrichment (NES = −1.398, FDR *q* = 0.104; Fig. 5A). To functionally validate this observation, we assessed the accumulation of K48-linked polyubiquitylated proteins. As expected, RAD23A-depleted MM cells exhibited a marked increase in K48-linked polyubiquitinated proteins across a panel of MM cell lines, indicating impaired clearance of ubiquitinated proteins (Fig. 5B). Excessive K48-linked polyubiquitination is also closely associated with aggresome formation [28]. Consistent with this, RAD23A-iKO cells displayed pronounced aggresome accumulation compared with control cells (Fig. 5C).

We next evaluated the effect of RAD23A depletion on proteasome catalytic activity in MM.1R RAD23A-iKO cells. The proteasome has three major activities: trypsin-like (TL), which cleaves after basic residues; chymotrypsin-like (CTL), which cleaves after hydrophobic residues; and caspase-like (CL), which cleaves after acidic residues. Our data showed that RAD23A depletion only affected CL activity, which increased upon depletion (Fig. 5D). This suggests that the accumulation of K48-linked polyubiquitinated proteins during RAD23A knockout is likely due to decreased shuttling of ubiquitinated proteins to the proteasome for degradation.

RAD23A depletion also modulates cellular stress response pathways. Proteomics-based GSEA revealed significant upregulation of the peroxisome (NES = 1.798, FDR *q* < 0.001) and apoptosis (NES = 1.354, FDR *q* = 0.124) pathways, accompanied by marked downregulation of mTORC1 signaling (NES = −1.951, FDR *q* < 0.001) (Data not shown). The peroxisome pathway is closely linked to reactive oxygen species (ROS) metabolism and lipid oxidative stress [29], while mTORC1 promotes anabolic growth and protein synthesis [30]. Immunoblotting further confirmed that RAD23A depletion notably increased stress response signaling, evidenced by increased phosphorylation of eIF2α and upregulation of ATF4 and CHOP (Fig. 5E). Together, these findings suggest that RAD23A loss shifts MM cells from a growth-promoting state toward a stress-adaptive and pro-death program.

Functionally, RAD23A depletion led to activation of the intrinsic apoptotic cascade, as evidenced by increased cleavage of caspase-3 and PARP (Fig. 5E). Consistent with this, caspase-3/7 enzymatic activity was significantly elevated in RAD23A-iKO cells compared with controls (Fig. 5F), and flow cytometric analysis confirmed a marked increase in apoptotic cell populations (Fig. 5G).

## Discussion

RAD23A occupies a unique biological position at the interface of DNA repair and ubiquitin-proteasome-mediated protein degradation [26]. Although its biochemical functions have been well characterized, its role in cancer biology has remained underexplored. Prior studies suggest that the consequences of RAD23A dysregulation are highly context dependent. In ovarian cancer, RAD23A expression is elevated in niraparib-resistant cells, where RAD23A promotes DNA repair following chemotherapy and contributes to resistance to PARP inhibitors, highlighting its role in DNA repair [31]. Conversely, a separate study in lung cancer cell lines showed that RAD23A depletion enhances autophagy, inhibits cell proliferation, and increases resistance to DNA-damaging agents, such as cisplatin and oxaliplatin [32], reflecting a context-dependent role of RAD23A in regulating the cellular stress response. Together, these observations indicate the complexity of RAD23A biology and suggest that its impact on therapy response is highly dependent on both tumor type and the dominant stress pathways engaged.

MM represents a particularly relevant setting in which to investigate RAD23A biology. MM cells are characterized by the clonal proliferation of plasma cells, profound proteotoxic stress, and an aberrant DNA damage response [1, 5, 33]. Finely tuned DNA repair capacity and proteasome-dependent degradation pathways are critical for MM cell survival [3, 34]. Our findings position RAD23A as a central coordinator of this balance. The association between elevated RAD23A expression and poor clinical outcome suggests that enhanced RAD23A function may provide advantages in the stress-adapted state characteristic of MM.

Mechanistically, our data support a model in which RAD23A integrates genome maintenance and proteostasis to assist MM cells against cumulative stress. Rather than functioning solely within one pathway, RAD23A appears to reinforce two complementary survival pathways - efficient resolution of DNA damage and clearance of poly-ubiquitinated proteins – with their relative importance potentially shifting depend on cellular stress conditions and binding partner availability. Disruption of RAD23A shifts this equilibrium, amplifying both genotoxic and proteotoxic stress signals. Beyond NER, RAD23A depletion also impaired double-strand break repair and homology-directed repair, indicating a broader role in the DNA damage response. Although RAD23A has previously been associated with proteasome enzymatic activities [35], we found that its depletion did not affect the TL and CTL activities, but increased CL activity. These results suggest that its primary contribution in MM may be to maintain substrate flux rather than directly regulate core proteolytic activities. The accumulation of ubiquitinated proteins and activation of stress pathways following RAD23A depletion indicate that impairing substrate shuttling alone is sufficient to destabilize proteostasis in MM. This aligns with our previous findings that targeting the subunits of the proteasome 19S regulatory particle, which operate upstream of the 20S catalytic core, can effectively block 26S proteasome function [26, 36–38]. Importantly, the proteasome is also known to participate in DNA repair process, particularly NER, where RAD23A has established functions. As a ubiquitin shuttle factor, RAD23A may contribute to these processes by linking ubiquitinated substrates at DNA damage sites to proteasome, thereby promoting efficient repair. Thus, RAD23A may support DNA repair through both its canonical role in DNA repair and its function in facilitating proteasome-related DNA damage repair.

From a therapeutic perspective, the dual role of RAD23A in DNA damage repair and proteasome-mediated protein degradation has important implications. Myeloma therapies commonly exploit proteotoxic stress using PIs or DNA damage using alkylators. A factor that simultaneously sustains DNA repair and proteostasis represents a strategic vulnerability. Inhibiting RAD23A could theoretically enhance the efficacy of established therapies and potentially overcome resistance mechanisms that rely on adaptive repair or stress pathways. The consistency of the observed effects across bortezomib-sensitive and -resistant settings further supports this possibility. Although selective small-molecule inhibitors of RAD23A are not yet established; RAD23A operates through structurally defined UBA and UBL interaction domains that mediate ubiquitin binding and proteasome docking. In addition, emerging RNA-based therapeutic platforms—including antisense oligonucleotides and RNA interference approaches—offer a feasible strategy to selectively reduce RAD23A expression, as genetic suppression has already demonstrated a functional and phenotypic impact in preclinical models.

In summary, our study identifies RAD23A as a stress-adaptive integrator that enables myeloma cells to tolerate the combined burden of genomic instability and proteotoxic load. By linking DNA repair capacity with proteasome-dependent protein degradation, RAD23A sustains a survival equilibrium that is critical for MM cells. These findings expand the conceptual framework of stress coordination in MM and nominate RAD23A as a potential therapeutic target. Future studies should evaluate pharmacologic strategies, alone or in combination with standard therapies, to determine whether targeting this dual-function protein can yield clinical benefit.

## Data Availability

For original data, please contact yan_song@dfci.harvard.edu.
